# Incidence and Predictors of Outcome in the Treatment of In-Stent Restenosis with Drug-Eluting Balloons, a Real-Life Single-Centre Study

**DOI:** 10.1155/2022/1395980

**Published:** 2022-08-29

**Authors:** Kyle Murnaghan, Helen Bishop, Navjot Sandila, Bakhtiar Kidwai, Lawrence Title, Ata Ur Rehman Quraishi, Catherine Kells, Hussein Beydoun, Osama Elkhateeb

**Affiliations:** Nova Scotia Health Authority, Halifax, Nova Scotia, Canada

## Abstract

**Objectives:**

To determine the one-year and five-year occurrence and prognosticators of major adverse cardiac events (MACE: composition of all-cause death, myocardial infarction, target vessel revascularization, and vessel thrombosis), mortality, and target lesion revascularization (TLR) in patients with in-stent restenosis (ISR) treated with drug-eluting balloons (DEBs).

**Background:**

DEBs have become an emerging therapeutic option for ISR. We report the results of a single-center retrospective study on the treatment of ISR with DEB.

**Methods:**

94 consecutive patients with ISR treated with the paclitaxel-eluting balloon were retrospectively studied between August 2011 and December 2019.

**Results:**

The one-year MACE rate was 11.8%, and the five-year MACE rate was 39.8%. The one-year mortality was 5.3%, and the five-year mortality rate was 21.5%. The one-year TLR rate was 4.3%, and the five-year rate was 18.7%. The univariable-Cox proportional hazard models for TLR showed lesion length, and the number of DEBs per vessel is associated with adverse outcomes with H.R. of 1.038 (1.007–1.069) and 4.7 (1.6–13.8), respectively.

**Conclusion:**

Our data indicate that at one year, DEBs provide an effective alternative to stenting for in-stent restenosis. Our five-year data, representing one of the longest-term follow-ups of DEB use, demonstrate high rates of MACE. The high five-year MACE reflects all-cause mortality in a high-risk population. This is offset by a reasonable five-year rate of TLR, indicating that DEB provides both short-term and long-term benefits in ISR.

## 1. Introduction

Interventional cardiology has dramatically changed outcomes for patients requiring coronary revascularization [1, 2]. However, this improvement has not been made without difficulty. As new therapies and stent technology have emerged, new challenges have followed, including in-stent restenosis (ISR). Drug-eluting balloons (DEBs) have emerged as a possible solution to the challenge of ISR.

Plain old balloon angiography (POBA) allowed the percutaneous intervention of stenosed coronary arteries. However, its efficacy was limited by elastic recoil and flow-limiting dissections. Bare metal stents (BMS) emerged as a solution, providing a support scaffold to the coronary artery, and reduced the immediate recoil, dissection, and recurrent intimal narrowing that plagued POBA. While there was an improvement over POBA, restenosis still occurred in vessels treated with BMS. By combining the support scaffold of BMS with an antiproliferative agent, drug-eluting stents (DES) emerged as a solution to restenosis [3]. While there was a significant improvement over BMS, a DES has not been an ideal solution for every patient with CAD [4]. The need for long-term dual antiplatelet therapy, the inability for stents to fit in small vessels, and in the case of in-stent restenosis, the addition of another layer of a stent can be undesirable [5].

In this regard, the treatment of DES-ISR has been particularly challenging. DES-ISR, such as BMS-ISR, is often due to underexpansion, stent misplacement, and stent fracture [6, 7]. However, unlike BMS-ISR, the effects of the antiproliferative agent in DES-ISR lead to focal and heterogeneous restenosis, with associated neoatherosclerosis and peri-strut inflammation. Furthermore, DES-ISR occurs later in the patient's treatment course than BMS-ISR, with a maximal late loss in BMS-ISR seen at 6–8 months, compared to ongoing late loss out to five years in DES-ISR [8]. It has been demonstrated that patients with DES-ISR have worse clinical outcomes than those with BMS-ISR [9]. These issues have led to the question of whether repeat stenting is the optimal solution to ISR. DEBs have emerged as a solution. By providing homogenous and high-dosed levels of antiproliferative therapy to the vessel wall, they reduce the inflammation, which leads to restenosis, without the added layer of stents and polymers [5]. This advantage was recognized in the 2014 European Society of Cardiology Myocardial Revascularization guidelines, where DEBs were recommended to treat both DES and BMS ISR [10].

Both DES and DEB have been proven in randomized controlled trials to be effective in BMS and DES-ISR. In a meta-analysis published by Yang et al. in the Journal of Interventional Cardiology, it was argued that DEB and DES provided uncertain outcomes for ISR treatment [9]. While the Restenosis Intra-stent of Drug-eluting Stents: Paclitaxel-eluting Balloon vs. Everolimus-eluting Stent (RIBS IV) study demonstrated the superiority of second-generation DES in DES-ISR at one and three years, and its applicability is limited by differences in trial design between the arms, length of follow-up, and high rate of bailout stenting [11]. Despite the proven benefits of DEB, there is limited real-world, all-comer data. Those that exist are limited to one-year clinical follow-up, and almost none have been completed in North America [12]. We present the use of DEBs in ISR treatment, with a specific focus on short-term and long-term outcomes. This study represents one of the most extensive North American studies on the use of DEBs in ISR and has a significant duration of follow-ups.

## 2. Methods

### 2.1. Patient Cohort

This retrospective study enrolled 94 consecutive patients treated with the paclitaxel-eluting balloon at the Queen Elizabeth II Health Sciences Centre (QEII) in Halifax, Nova Scotia, between August 2011 and December 2019. Patients were eligible for the study if they were ≥ 18 years old and had received a coronary intervention with a paclitaxel-eluting balloon during the investigation timeframe. Those enrolled represented every patient treated at the QEII with a DEB during this period.

The institutional research ethics board approved this study. Data included in the study came from the cardiovascular health information system database, which is compiled of patients treated in the cardiac catheterization laboratory at the QEII Health Sciences Centre. This database includes patient demographics, procedural complications, devices used, and procedural outcomes. A manual chart review was conducted to complete missing data and review the need for repeat revascularization procedures. The catheterization laboratory at the QEII Health Science Centre in Halifax is the only catheterization lab in the province of Nova Scotia, which allows these data to capture all potential repeat catheterizations or revascularizations. All repeat catheterization angiograms were reviewed to identify any adverse outcomes.

### 2.2. Endpoints

Endpoints were major adverse cardiac events (MACE) which are defined as a composition of all-cause death, myocardial infarction, target vessel revascularization (TLR), vessel thrombosis, and the individual components of MACE at one and five years.

### 2.3. Clinical Data

Clinical data included baseline patient characteristics, history, and indications for the procedure. Procedural data included the intervention stage, target vessel, peri-procedural, and post-procedural therapies. Angiographic data included lesion size pre-intervention and post-intervention, including thrombolysis in myocardial infarction (TIMI) flow. Follow-up data included mortality, repeat angiography, TLV, TLR, ISR, and the indication for repeat intervention. ISR was defined as an angiographic luminal narrowing of > 50% diameter in-stent stenosis or within 5 mm of a stent.

### 2.4. Statistical Analysis

Baseline demographics for the study population were summarized as the mean (standard deviation) or median (interquartile range) for continuous variables and frequency (percent) for categorical variables. The outcomes of MACE and mortality were characterized using Kaplan–Meier plots, and the outcome of TLR was characterized using the cumulative incidence function. A competing risks analysis was used for TLR, with death before TLR considered a competing risk. One-year and five-year survival rates were calculated.

The general model selection approach outlined by Collet was used to fit Cox proportional hazards regression models for the outcomes of time to MACE and mortality and to fit cause-specific hazard models for time to TLR [13]. First, univariable models for each predictor of interest were fit, and those significant at a level of 0.20 were identified. Then, a multivariable model with all significant univariable predictors was fit using automatic backward selection, and those predictors nonsignificant at the level of 0.10 were eliminated. Nonsignificant predictors from the univariable analysis were then considered for the model using automated forward selection at a significance level of 0.10. Lastly, automated stepwise selection at the significance level of 0.05 was used to produce the final multivariable model. The univariable and multivariable models were summarized using the hazard ratios (H.R.), 95% confidence intervals, and corresponding *p* values. Firth's penalized maximum likelihood bias reduction method was used in cases of nonconvergence of the likelihood function.

The general model selection approach outlined by Collet was used to fit Cox proportional hazards regression models for the outcomes of time to MACE and mortality and to fit cause-specific hazard models for time to TLR [13]. First, univariable models for each predictor of interest were fit, and those significant at a level of 0.20 were identified. Then, a multivariable model with all significant univariable predictors was fit using automatic backward selection, and those predictors nonsignificant at the level of 0.10 were eliminated. Nonsignificant predictors from the univariable analysis were then considered for the model using automated forward selection at a significance level of 0.10. Lastly, automated stepwise selection at the significance level of 0.05 was used to produce the final multivariable model. The univariable and multivariable models were summarized using the hazard ratios (H.R.), 95% confidence intervals, and corresponding *p* values. Firth's penalized maximum likelihood bias reduction method was used in cases of nonconvergence of the likelihood function.

A two-sided *p* value of <0.05 was the threshold for statistical significance unless otherwise specified. All analyses were performed using SAS statistical software version 9.4 (SAS Institute Inc., Cary, N.C., USA) or *R* version 3.6.1 using the *R* package “coxphf.”

## 3. Results

This study examined 114 lesions in 94 patients treated with the paclitaxel-eluting balloon. The cohort's mean age was 65.5+/9.7 years, and 68.1% (64) were males. The demographic data are shown in Table 1. ISR represented 96.3% (88) of cases, 84.6% (77) were ISR following DES, and 11.7% (11) were ISR following BMS. DEB was utilized in 10.6% (10) of cases to treat de novo graft disease. ST-elevation myocardial infarction (STEMI) was the presentation of 4.3% (4) of patients, 25.5% (24) presented as a non-ST elevation myocardial infarction (NSTEMI), 41.5% (39) presented as unstable angina, and 25.5% (24) as stable angina. The indication for the procedures is shown in Table 2.

The most common vessel intervened upon was the right coronary artery (RCA) at 34% (32), followed by left anterior descending (LAD) at 26.6% (25) and the circumflex (Cx) at 22.3% (21). A saphenous vein graft (SVG) was intervened upon in 10.6% (10) cases. Of the lesions, 9.6% (9) of were completely occluded, 80.9% (76) were >70% stenosed, 7.5% (7) were 50 to 70% stenosed, and 2.1% (2) were <50% stenosed. Intravascular imaging (IVUS/OCT) was used in 20.2% (19) of lesions. The mean balloon length was 22.2 mm (SD of 9.1), and the median balloon length was 20 mm (IQR 15, 25). In 97.9% (92) of cases, the outcome was deemed procedurally successful by the operator. The procedural data are presented in Table 3.

**Figure 1:**
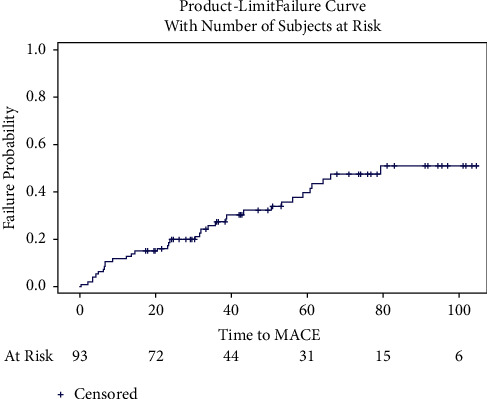
Time to MACE.

**Figure 2:**
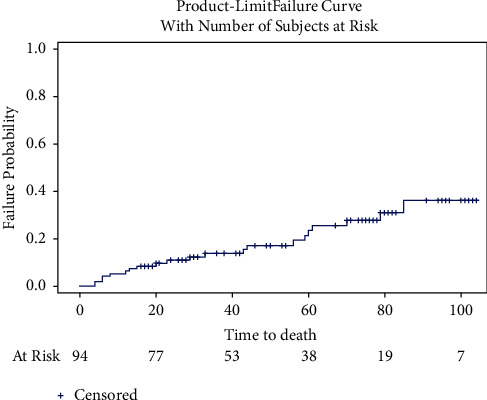
Time to mortality.

**Figure 3:**
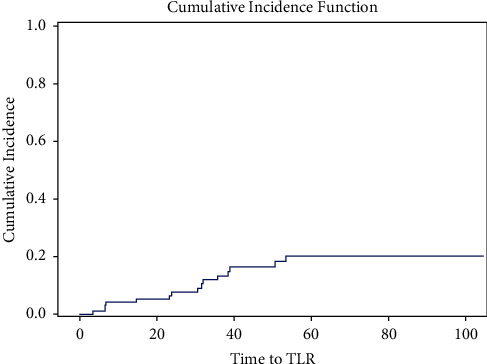
Time to TLR.

In total, 81.9% (77) of patients were discharged on clopidogrel and 17% (16) on ticagrelor. Almost all patients were discharged on aspirin, at 98.9% (93). The postprocedural data are presented in Table 4. The median follow-up was 37.5 months (IQR of 20, 71). The mean follow-up was 45.2 months (SD 30.6). One-year mortality was 5.3%, and five-year mortality was 21.5%. The one-year MACE rate was 11.8%, and the five-year MACE was 39.8%. Total TLR was 15% (14), with the one-year rate being 4.3% and the five-year rate being 18.7%. The one-year and five-year outcome data are presented in Table 5. The Kaplan–Meier curves of MACE and mortality are presented in Figures 1 and 2. The cumulative incident curves for TLR are represented in Figure 3. The univariable and multivariable Cox proportional hazard (H.R.) models for MACE, mortality, and TLR are presented below their corresponding figures, respectively, titled Tables 6–8.

## 4. Discussion

This is a large real-world all-comers study on DEB use in CAD in North America, with a significant duration of clinical follow-up. Our study showed that clinical outcomes in DEB for treatment of ISR are reasonable with lesion length and the number of balloons used as a risk for TLR.

The use of DEBs was evaluated previously in RIBS IV and restenosis intra-stent of bare metal stents: paclitaxel-eluting balloon vs. everolimus-eluting stent (RIBS V) trials. Although, the short-term one-year outcomes were inferior to DES, they did provide a reasonable alternative with mortality and a MACE rate of 1.9% and 18%, respectively. Our study's mortality at one year was higher (5.3%), indicating a potentially more comorbid population with more diabetes and previous myocardial infarction. Our one-year MACE was lower (11.8%) with lower TLR rates that are similar to the TLR rates in the DES arm of the RIBS trial (4.5%) [14]. This demonstrated that our study has comparable cardiac outcomes to the RIBS-DEB arm.

When comparing the demographic data of the RIBS DEB and DES arm to our study, they were similar in age; however, there were considerably more comorbidities in our study in terms of diabetes, dyslipidemia, and hypertension. The presentation was also different, with roughly half of RIBS patients presenting with stable angina, compared to only 25.5% in our cohort with the remainder presenting as acute coronary syndrome (ACS). Furthermore, the vessel intervened upon in RIBS was most commonly the LAD, followed by the RCA, LCx, and then SVG [14]. This is in contrast to our experience where the most commonly intervened upon vessels were the RCA, followed by the LAD, LCX, SVG, and then the left main.

While RIBS IV demonstrated the superiority of second-generation DES in DES-ISR, our study demonstrated results that more closely represented their DES arm. Our one-year TLR rate of 4.3% compares very favourably to RIBS IVs DES arm's one-year TLR rate of 4.5% [15]. Interestingly, this was not reflected in RIBS V which compared DES and DEB in BMS-ISR, as their one-year TLR rate was 1% in the stent group and 6% in the DEB group [16]. This indicates that patients with DES-ISR may benefit more from DEB administration than those with BMS-ISR.

There have been few studies that compared the five-year outcomes of DEBs in ISR vs. DES. Miura et al.'s retrospective study had a five-year outcome similar to ours with a mortality rate of 18.3%, compared to 21.5% in our cohort; their five-year MACE was 47.7% compared to this study's MACE at 39.8%. Finally, a five-year TLR of 34.1% in Miura's study was compared to our cohort's five-year TLR of 18.7% [17]. This demonstrates that our patient's cohort, while more comorbid, had significantly better five-year rates of TLR.

The key procedural findings of this study were that DEB angioplasty in ISR provides acceptable results in an otherwise considerably comorbid population. Furthermore, the key determinant of TLR in patients treated with DEB at our institution was balloon length and the number of balloons used per vessel. On univariable analysis, total lesion length had a cause-specific H.R. of 1.038 (*p*0.0149, 95% CI 1.007–1.069). This was also seen in the univariable analysis of the number of DEBs per vessel, where >1 had a cause-specific H.R. of 4.689 (*p*0.0049, 95% CI 1.596–13.77). In the multivariable analysis, the total lesion length became insignificant. However, DEBs per vessel remained significant at 3.986 (p 0.0124, 95% CI 1.349–11.781). This suggests that long diffuse lesions, particularly those treated with multiple DEB, are at the highest risk for failure. This is a prognostic indicator which previous studies may have underappreciated, potentially suggesting limiting the use of DEBs to focal lesions where only a single DEB is required.

On univariable analysis, this study demonstrated that the key determinate of MACE in our population was age with an H.R. of 1.045 (*p* of 0.02, 95% CI 1.006–1.085) and a past medical history of CABG with an H.R. of 2.4 (*p* of 0.01, CI 1.232–4.704). These results persisted in a multivariable analysis where the H.R. for age was 1.039 (*p*0.045, CI 1.001–1.079) and H.R. for CABG was 2.217 (*p*0.021, CI 1.27–4.361). While unsurprising, these data may help identify which patients would be best served by DEBs vs. DES in ISR.

### 4.1. Study Limitations

This study's fundamental limitations are secondary to its retrospective design and size. As a retrospective study, the role that selection bias plays cannot be excluded. Furthermore, the small size in terms of intervention upon de novo lesions limits commentary due to insufficient statistical significance. Despite these limitations, this study reflects patients treated consecutively by experienced interventional cardiologists. Doing so provides meaningful clinical data for both clinicians and patients.

## 5. Conclusions

This single-centre, real-world retrospective study demonstrates low rates of TLR at one year and moderate levels at five years. These data support the use of the DEB in ISR as a safe and efficacious treatment with a reasonable MACE rate, which was driven mainly by all-cause death in a comorbid patient population. Furthermore, this study demonstrates that balloon length and the need for multiple balloons are poor procedural prognostic markers. It also shows a significant risk of MACE and death with both age and history of bypass surgery. This information should help clinicians make informed decisions when selecting which cases of ISR are treated with the DEB.

## Figures and Tables

**Table 1:** Demographic and baseline characteristics.

Demographic and baseline characteristics	Total population *n* = 94
Age, mean (sd)	65.5 (9.7)
Age, median (IQR)	66 (59, 72)
Sex, male, *n* (%)	64 (68.1)
Diabetes mellitus, *n* (%)	56 (59.6)
Hypertension, *n* (%)	89 (94.7)
Dyslipidemia, *n* (%)	90 (95.7)
Smoking, *n* (%)	33 (35.1)
Prior PCI, *n* (%)	93 (98.9)
Prior MI, *n* (%)	73 (77.7)
Prior CABG, *n* (%)	31 (33)
Kidney disease, *n* (%)	21 (22.3)

**Table 2:** Indication for procedure.

Indication for procedure	
STEMI, *n* (%)	4 (4.3)
NSTEMI, *n* (%)	24 (25.5)
Unstable angina, *n* (%)	39 (41.5)
Stable angina, *n* (%)	24 (25.5)
Other, *n* (%)	3 (3.2)
ISR-DES, *n* (%)	77 (84.6)
ISR-BMS, *n* (%)	11 (11.7)
Graft disease, *n* (%)	10 (10.6)

**Table 3:** Procedural data.

Procedural data	
Lesion prestenosis (%)	
<50%, *n*	2 (2.1)
50–70%, *n*	7 (7.5)
>70%, *n*	76 (80.9)
100%, *n*	9 (9.6)
Pre-TIMI 3flow, *n* (%)	71 (75.5)
Post-TIMI 3flow, *n* (%)	93 (98.9)

Interventional stage (%)	
Pretreatment, *n* (%)	3 (3.2)
Primary, *n* (%)	80 (85.1)
Adjunct, *n* (%)	7 (7.4)
Missing, *n* (%)	4 (4.3)

Target vessel (%)	
Cx, *n* (%)	21 (22.3)
LAD, *n* (%)	25 (26.6)
LMS, *n* (%)	6 (6.4)
RCA, *n* (%)	32 (34)
SVG, *n* (%)	10 (10.6)
ATM pressure in mmHg, mean (sd)	65.5 (9.7)
ATM pressure in mmHg, median (IQR)	66 (59, 72)

Number of vessel PCI	
1 vessel PCI, *n* (%)	81 (86.2)
2 vessel PCI, *n* (%)	12 (12.8)
3 vessel PCI, *n* (%)	1 (1.1)
Bifurcation, *n* (%)	13 (13.8)
GP iiB/iiiA use, *n* (%)	2 (2.1)
Bivalirudin use, *n* (%)	23 (24.5)
OCT/IVUS, *n* (%)	19 (20.2)
Angiographic failure, *n* (%)	2 (2.1)
Angiographic success, *n* (%)	92 (97.9)
Number of lesions, mean (sd)	1.2 (0.5)
Number of lesions, median (IQR)	1 (1, 1)

Number of lesions (%)	
1 *n*, (%)	82 (87.2)
≥2 *n*, (%)	12 (12.8)
Length of balloon in mm, mean (sd)	22.2 (9.2)
Length of balloon in mm, median (IQR)	20 (15, 25)

**Table 4:** Postprocedural data.

Postprocedural data	Total population *n* = 94
ASA on discharge, *n* (%)	93 (98.9)
Clopidogrel on discharge, *n* (%)	77 (81.9)
Ticagrelor on discharge, *n* (%)	16 (17)

**Table 5:** Results.

Outcome	1-year (95% CI)	5-year (95% CI)
MACE	11.8% (0.067, 0.203)	39.8% (0.293, 0.525)
Mortality	5.3% (0.023, 0.123)	21.5% (0.134, 0.334)
TLR	4.3% (0.014, 0.098)	18.7% (0.106, 0.286)

**Table 6:** Univariable and multivariable Cox proportional hazard (H.R.) for MACE.

Variable	Univariable		Multivariable model	
	H.R. (95% CI)	*p* HR	HR (95% CI)	*p* H.R.
Age	1.045 (1.006, 1.085)	0.0227	1.039 (1.001, 1.079)	0.0456
Total balloon length in mm	1.015 (0.989, 1.042)	0.2577		
Number of DEBs per vessel >1	2.068 (0.901, 4.748)	0.0867		
Sex, female	1.494 (0.758, 2.944)	0.2459		
Vessel lesion		0.3261		
Cx (ref)				
LAD	0.957 (0.321, 2.85)	0.9369		
LMS	2.502 (0.623, 10.051)	0.1963		
RCA	1.607 (0.616, 4.191)	0.3321		
SVG	2.601 (0.784, 8.628)	0.1181		
Hypertension	2.629 (0.36, 19.219)	0.341		
Diabetes	1.739 (0.85, 3.557)	0.1295		
Smoker	0.912 (0.446, 1.865)	0.8005		
Dyslipidemia	2.031 (0.278, 14.859)	0.4852		
Prior MI	1.373 (0.598, 3.154)	0.4552		
Prior CABG	2.407 (1.232, 4.704)	0.0102	2.217 (1.127, 4.361)	0.0211
Graft failure	0.868 (0.379, 1.989)	0.7384		
ISR-DES	1.61 (0.566, 4.582)	0.3724		
ISR-BMS	0.575 (0.175, 1.883)	0.3603		

**Table 7:** Univariable and multivariable Cox proportional hazard (H.R.) for mortality.

Variable	Univariable		Multivariable model	
	H.R. (95% CI)	*p* HR	HR (95% CI)	*p* H.R.
Age	1.103 (1.046, 1.163)	0.0003		
Total lesion length in mm	0.978 (0.925, 1.034)	0.4273		
Number of DEB per vessel >1	1.278 (0.376, 4.346)	0.695		
Sex, female	1.193 (0.492, 2.893)	0.6959		
Vessel lesion		0.0772		
Cx (ref)				
LAD	0.598 (0.16, 2.229)	0.4437		
LMS	2.945 (0.698, 12.432)	0.1416		
RCA	0.547 (0.158, 1.892)	0.3406		
SVG	2.191 (0.577, 8.313)	0.2492		
Hypertension	1.497 (0.2, 11.206)	0.6944		
Diabetes	1.971 (0.759, 5.118)	0.1633		
Smoking	0.635 (0.245, 1.646)	0.3502		
Dyslipidemia	1.078 (0.144, 8.048)	0.9415		
Prior MI	1.937 (0.569, 6.595)	0.29		
Prior CABG	2.829 (1.193, 6.71)	0.0183		
Renal failure	1.061 (0.388, 2.901)	0.9084		
ISR-DES	1.35 (0.393, 4.644)	0.6338		
ISR-BMS	0.563 (0.13, 2.433)	0.4415		

**Table 8:** Univariable and multivariable cause-specific Cox proportional hazard (H.R.) for TLR.

Variable	Univariable		Multivariable model	
	H.R. (95% CI)	*p* HR	HR (95% CI)	*p* H.R.
Age	1.019 (0.964, 1.077)	0.4399		
Total lesion length in mm	1.038 (1.007, 1.069)	0.0149		
Number of DEB per vessel >1	4.689 (1.597, 13.77)	0.0049	3.986 (1.349, 11.781)	0.0124
Sex, female	1.116 (0.381, 3.27)	0.841		
Vessel lesion		0.0732		
Cx (ref)				
LAD	1.311 (0.174, 14.334)	0.7113		
LMS	1.739 (0.012, 32.741)	0.7473		
RCA	3.339 (0.752, 31.376)	0.1441		
SVG	8.905 (1.621, 89.77)	0.0277		
Hypertension	2.209 (0.298, 282.12)	0.5303		
Diabetes	0.834 (0.302, 2.3)	0.7253		
Smoking	1.745 (0.632, 4.813)	0.2824		
Dyslipidemia	1.886 (0.254, 240.881)	0.6245		
Prior MI	2.187 (0.492, 9.715)	0.3036		
Prior CABG	3.875 (1.371, 10.952)	0.0106	3.443 (1.21, 9.793)	0.0205
Renal failure	0.859 (0.242, 3.046)	0.814		
ISR-DES	3.071 (0.403, 23.378)	0.2787		
ISR-BMS	0.414 (0.054, 3.153)	0.3946		

## Data Availability

Data are available on request by contacting the corresponding author.

## Abbreviations

PCI: Percutaneous coronary intervention
ISR: In-stent restenosis
DCB: Drug-coated balloons
DEB: Drug-eluting balloons.
